# Toward Active Distributed Fiber-Optic Sensing: A Review of Distributed Fiber-Optic Photoacoustic Non-Destructive Testing Technology

**DOI:** 10.3390/s26010059

**Published:** 2025-12-21

**Authors:** Yuliang Wu, Xuelei Fu, Jiapu Li, Xin Gui, Jinxing Qiu, Zhengying Li

**Affiliations:** 1 School of Information Engineering, Wuhan University of Technology, Wuhan 430062, China; yuliangwu@whut.edu.cn (Y.W.);; 2 National Engineering Research Center of Fiber Optic Sensing Technology and Networks, Wuhan University of Technology, Wuhan 430062, China; 3 State Key Laboratory of Advanced Technology for Materials Synthesis and Processing, Wuhan University of Technology, Wuhan 430062, China

**Keywords:** distributed fiber-optic photoacoustic transducer array, fiber-optic ultrasonic sensing, photoacoustic materials, non-destructive testing, structural health monitoring

## Abstract

Distributed fiber-optic photoacoustic non-destructive testing (DFP-NDT) represents a paradigm shift from passive sensing to active probing, fundamentally transforming structural health monitoring through integrated fiber-based ultrasonic generation and detection capabilities. This review systematically examines DFP-NDT’s evolution by following the technology’s natural progression from fundamental principles to practical implementations. Unlike conventional approaches that require external excitation mechanisms, DFP-NDT leverages photoacoustic transducers as integrated active components where fiber-optical devices themselves generate and detect ultrasonic waves. Central to this technology are photoacoustic materials engineered to maximize conversion efficiency—from carbon nanotube-polymer composites achieving 2.74 × 10^−2^ conversion efficiency to innovative MXene-based systems that combine high photothermal conversion with structural protection functionality. These materials operate within sophisticated microstructural frameworks—including tilted fiber Bragg gratings, collapsed photonic crystal fibers, and functionalized polymer coatings—that enable precise control over optical-to-thermal-to-acoustic energy conversion. Six primary distributed fiber-optic photoacoustic transducer array (DFOPTA) methodologies have been developed to transform single-point transducers into multiplexed systems, with low-frequency variants significantly extending penetration capability while maintaining high spatial resolution. Recent advances in imaging algorithms have particular emphasis on techniques specifically adapted for distributed photoacoustic data, including innovative computational frameworks that overcome traditional algorithmic limitations through sophisticated statistical modeling. Documented applications demonstrate DFP-NDT’s exceptional versatility across structural monitoring scenarios, achieving impressive performance metrics including 90 × 54 cm^2^ coverage areas, sub-millimeter resolution, and robust operation under complex multimodal interference conditions. Despite these advances, key challenges remain in scaling multiplexing density, expanding operational robustness for extreme environments, and developing algorithms specifically optimized for simultaneous multi-source excitation. This review establishes a clear roadmap for future development where enhanced multiplexed architectures, domain-specific material innovations, and purpose-built computational frameworks will transition DFP-NDT from promising laboratory demonstrations to deployable industrial solutions for comprehensive structural integrity assessment.

## 1. Introduction

In the intricate network of modern industrial systems, large-scale equipment plays an indispensable and central role [1]. The safety, stability, and reliability of such equipment are fundamental not only to national economic infrastructure but also to public safety and the sustainability of the ecological environment. Systems such as commercial aircraft, long-distance oil and gas pipelines, offshore cargo vessels, and large chemical storage tanks typically operate under harsh conditions, enduring prolonged exposure to dynamic loads, corrosive environments, and material fatigue [2,3,4]. As operational time accumulates, these structures are prone to various types of structural damage. The evolution of such damage directly affects the functional integrity and safety margins of the entire system, representing a persistent and significant challenge in engineering [5].

**Figure 1:**
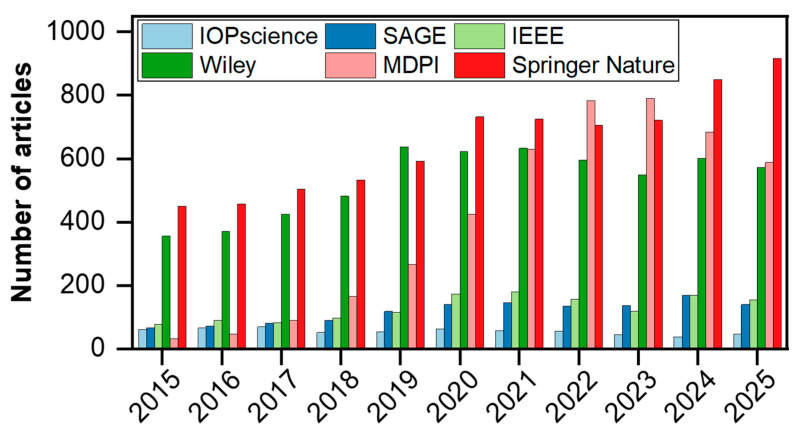
Number of publications by each publisher in non-destructive testing related papers from “web of science” statistical data over the past decade.

Structural damage in large-scale equipment is often highly concealed, arises randomly in both location and time, develops rapidly, and poses severe risks—making it the greatest threat to safe and stable operation [6,7,8], as illustrated in Figure 1. For instance, in 2002, the China Airlines flight 611 crash was attributed to a latent scratch-induced crack on the aircraft’s fuselage skin, which rapidly propagated during flight into catastrophic structural failure, resulting in the disintegration of the plane and the tragic loss of all 228 passengers on board [9]. In 2018, a propylene pipeline rupture at a construction site in Nanjing went undetected, leading to gas dispersion and a massive subsequent explosion that claimed 22 lives [10]. In 2020, the grounding of the bulk carrier MV Wakashio caused hull fractures and fuel leakage; the spill was not identified for several days, leading to severe ecological and economic consequences for Mauritius [11]. The same year, a styrene storage tank ruptured in India due to undetected structural flaws, causing a toxic gas leak that killed 10 people and affected over 5000 residents [12]. In 2021, an aging offshore oil pipeline off the coast of California ruptured; nearly 480,000 L of crude oil had already entered the Pacific Ocean before the leak was discovered, triggering a major ecological disaster [13]. These high-profile incidents collectively highlight that undetected structural damage can evolve unchecked and rapidly during equipment operation, ultimately culminating in catastrophic failures with significant loss of life and economic damage. Therefore, there is an urgent need for a monitoring method capable of continuous, full-coverage inspection during equipment operation—one that can promptly detect damage locations, provide early warning of damage propagation, and prevent accidents, thereby ensuring the safe and reliable operation of large-scale systems.

Non-destructive testing (NDT) techniques enable the detection of early-stage structural flaws without impairing the integrity of the inspected component. By providing information on the size, location, nature, and quantity of damage, NDT allows for accurate assessment of the structural health state of equipment [14,15]. Among current mainstream NDT methods, ultrasonic testing (UT), eddy current testing, and radiographic testing are widely adopted [16,17,18]. Ultrasonic testing, in particular, excites ultrasonic waves using transducers and detects signals altered by interaction with structural defects. By analyzing these signals, it identifies flaw characteristics and locates damage with high precision. Owing to its advantages of high damage sensitivity, deep penetration capability, and environmental friendliness, ultrasonic NDT is the most extensively applied technique [18,19,20,21]. Over the past decade, the number of publications related to ultrasonic NDT has steadily increased across major publishers (Figure 1), indicating that this field still holds significant potential for further exploration and innovation.

Piezoelectric ultrasonic testing, which employs piezoelectric ceramics as transducers for contact-based inspection, offers high excitation efficiency, high detection sensitivity, and fast response. However, it requires point-by-point cabling for both excitation and sensing, significantly increasing system weight and complexity. This makes it extremely difficult to achieve full-area coverage on large-scale equipment [22,23,24,25]. Laser ultrasonic testing, on the other hand, generates and detects ultrasonic remotely using lasers without physical contact or extensive cabling. Nevertheless, due to the limitations of free-space optical coupling, the irradiation range of a single laser unit is restricted. Achieving integrated, large-area inspection would necessitate the deployment of numerous laser units, which would greatly increase system load, reduce equipment mobility, and compromise operational flexibility [26,27]. Consequently, there is a pressing demand for a lightweight, scalable ultrasonic transducer array that can be integrated into equipment with minimal burden, enabling wide-coverage, distributed excitation and detection. Such a system would form the basis for a large-area, distributed, and real-time ultrasonic NDT solution—capable of “feeling” structural health conditions in real time and safeguarding the reliable operation of complex engineering systems.

**Figure 2:**
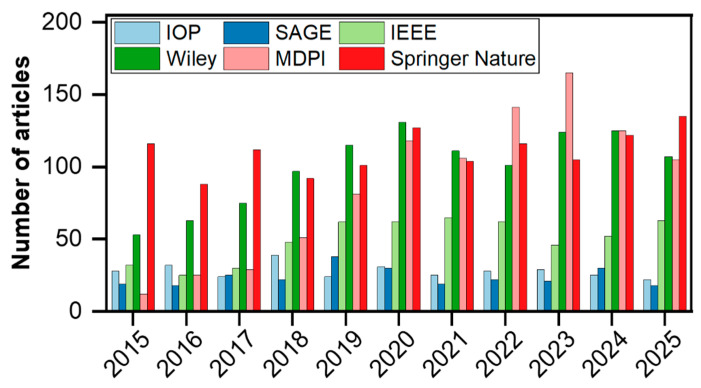
Number of publications on fiber-optic photoacoustics by each publisher from “web of science” statistical data over the past decade.

To address these challenges, distributed fiber-optic photoacoustic sensing (DFPS) has emerged as a promising alternative [28]. This approach transforms optical fiber sensing from passive sensing to active probing. By leveraging distributed fiber-optic photoacoustic transducers, this technology enables lightweight, broad-area ultrasonic generation and detection, offering inherent advantages for continuous, in situ structural health monitoring. In the past decade, research on fiber-optic photoacoustic NDT has witnessed rapid growth, especially over the last five years, with a significant increase in the number of publications across major publishers (Figure 2), reflecting its rising importance and technical maturity.

As illustrated in Table 1, DFP-NDT fundamentally redefines the system architecture of ultrasonic NDT by integrating both excitation and detection within a single, lightweight, all-fiber platform. Unlike piezoelectric systems burdened by electrical cabling and electromagnetic interference (EMI), or laser ultrasonics constrained by free-space optical coupling and high equipment cost, DFP-NDT enables cable-free, EMI-immune, and conformally deployable structural interrogation—marking a true paradigm shift toward scalable, real-time, and embedded structural health monitoring.

This review presents a systematic and multidimensional analysis of distributed fiber-optic photoacoustic non-destructive testing (DFP-NDT), organized around six core themes that define its technological ecosystem. First, we examine the photoacoustic materials, focusing on light-to-sound conversion mechanisms and performance metrics. Second, we analyze the microstructural designs of single-point fiber-optic photoacoustic transducers (FOPTs) that enable directional, broadband, or high-temperature operation. Third, we classify the six principal methodologies for constructing distributed fiber-optic photoacoustic transducer arrays (DFOPTAs)—side wall leakage, core-offset splicing, fiber splicing, tapering or collapsing, grating structures, and multi-channel systems—and assess their trade-offs in scalability and signal uniformity. Fourth, we critically evaluate the imaging algorithms adapted or developed for DFP-NDT data, including Total Focusing Method (TFM), RAPID, and AI-based approaches. Fifth, we summarize real-world applications across metal, composite, and corrosion monitoring scenarios, demonstrating operational versatility. Finally, we identify persistent technical challenges and emerging trends, offering a forward-looking perspective on material innovations, scalable architectures, and domain-specific computational frameworks. By following this structured progression—from physical principles to system integration and practical deployment—this work establishes a comprehensive roadmap for advancing DFP-NDT from lab-scale prototyping to industrial implementation.

## 2. Fiber-Optic Ultrasonic NDT

Distributed fiber-optic sensing has matured into a powerful platform for structural health monitoring, with established techniques including Brillouin, Raman, and Rayleigh scattering-based methods for distributed temperature and strain sensing [29,30,31,32,33]. Within this ecosystem, fiber-optic ultrasonic detection represents a critical modality for high-resolution defect diagnosis.

**Figure 3:**
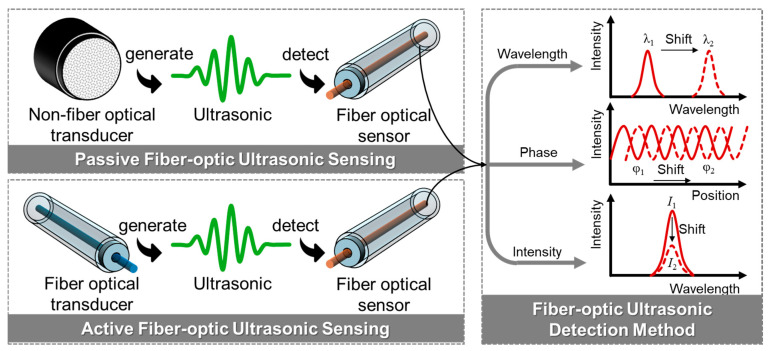
Passive fiber-optic ultrasonic method and active fiber-optic ultrasonic method.

Fiber-optic ultrasonic NDT can be classified into passive and active configurations based on whether the ultrasonic is generated by the fiber-optical transducer, as illustrated in Figure 3. In passive fiber-optic ultrasonic NDT, ultrasonic waves are generated externally—typically by piezoelectric transducers or through acoustic emissions originating from structural damage—and subsequently detected by fiber-optic ultrasonic sensors [34,35,36,37]. In contrast, active fiber-optic ultrasonic NDT employs a fiber-based transducer to generate ultrasonic waves, while fiber-optic sensors are used for detection. In both configurations, the incident ultrasonic waves induce dynamic perturbations in the fiber sensor, leading to changes in its optical characteristics—such as wavelength, phase, or intensity [38]. By demodulating these optical signal variations, the original ultrasonic waveform can be reconstructed and analyzed for defect identification and localization. Table 2 summarizes the key architectural distinctions between passive and active configurations, underscoring how DFP-NDT achieves functional integration through opto-thermo-mechanical energy conversion at the fiber interface.

### 2.1. Passive Fiber-Optic Ultrasonic NDT

#### 2.1.1. Principle of Fiber-Optic Ultrasonic Detection

As representative examples of typical fiber-optic ultrasonic sensors, fiber Bragg gratings (FBGs) and Fabry–Pérot interferometers (FPIs) operate based on distinct physical principles for ultrasonic detection.

The core mechanism of FBG-based ultrasonic sensing lies in the strain-induced shift in the Bragg wavelength $\lambda_{B}$. The Bragg wavelength is determined by [39,40]:

(1) $$\lambda_{B}=2n_{\mathrm{eff}}\Lambda$$

where $n_{\mathrm{eff}}$ denotes the effective refractive index of the fiber core and Λ represents the grating period. When an axial dynamic strain ε(t) is induced by an incident ultrasonic wave, the corresponding wavelength shift $\Delta \lambda_{B}$ follows [39,40]:

(2) $$\frac{\Delta \lambda_{B}}{\lambda_{B}}=\left(1-p_{e}\right)\varepsilon \left(t\right)$$

where $p_{e}$ is the photoelastic coefficient, which accounts for both geometric deformation and refractive index change due to strain. An ultrasonic longitudinal wave propagating through the medium generates a time- and space-dependent strain field given by [39,40]:

(3) $$\varepsilon \left(x,t\right)=\frac{1}{E}\sigma \left(x,t\right)=\frac{P_{0}}{E}\sin\left(kx-\omega t+\phi \right)$$

where $P_{0}$ is the acoustic pressure amplitude, E is the Young’s modulus of the fiber, $k=2\pi f/c_{s}$ is the wavenumber, f is the ultrasonic frequency, $c_{s}$ is the speed of sound in the medium, and ϕ is a phase constant. Substituting Equation (3) into Equation (2), the time-dependent Bragg wavelength shift becomes [41]:

(4) $$\Delta \lambda_{B}\left(t\right)=\lambda_{B}\left(1-p_{e}\right)\frac{P_{0}}{E}\sin\left(2\pi ft+\phi \right)$$

This indicates a linear relationship between the Bragg wavelength shift and the acoustic pressure amplitude, enabling quantitative ultrasonic measurement. However, when considering the finite grating length $L_{g}$ of the FBG, spatial averaging must be considered if the ultrasonic wavelength $\Lambda_{s}$ is comparable to or smaller than $L_{g}$. In this case, the effective strain sensed by the FBG is integrated over its entire length:

(5) $$\Delta \lambda_{B}\left(t\right)=\lambda_{B}\left(1-p_{e}\right)\frac{1}{L_{g}}\int_{0}^{L_{g}}\varepsilon \left(x,t\right)dx$$

When $\Lambda_{s}\ll L_{g}$, this integration leads to significant signal averaging, reducing the sensor’s response. Conversely, when $\Lambda_{s}\gg L_{g}$, the FBG responds primarily to the local peak strain. Since typical ultrasonic wavelengths (on the order of millimeters or less) are often much smaller than practical FBG lengths (e.g., several millimeters to centimeters), Equation (5) is more representative in practice. Consequently, longer gratings result in lower sensitivity due to spatial averaging—a key trade-off between sensitivity and reflectance in FBG-based ultrasonic detection.

The fundamental principle of FPI-based ultrasonic detection involves the modulation of the cavity length ΔL(t) in response to acoustic pressure p(t). For an intrinsic FPI, the incident ultrasonic wave directly compresses the cavity, altering its optical path length. The transmitted optical intensity is described by [42,43,44]:

(6) $$I=I_{0}\frac{1}{1+F\sin^{2}\left(\frac{\delta }{2}\right)},\delta =\frac{4\pi nL}{\lambda }+\phi_{\mathrm{ext}}$$

where n is the refractive index of the cavity medium, L is the static cavity length, F is the finesse of the interferometer, λ is the probe wavelength, and $\phi_{\mathrm{ext}}$ represents additional phase perturbations introduced by external stimuli. The acoustic pressure p(t) induces a change in cavity length ΔL(t). For a cavity with effective end-face area A, this can be expressed as [42,43,44]:

(7) $$\Delta L\left(t\right)=\frac{P\left(t\right)\cdot A\cdot L_{0}}{EA}=\frac{P\left(t\right)L_{0}}{E}$$

where $P(t)=P_{0}sin(\omega t)$ is the time-varying acoustic pressure, and $L_{0}$ is the initial cavity length. Under small-signal conditions and operation within the linear range of the interferometer, the output intensity modulation is approximately proportional to ΔL(t) [42,43,44]:

(8) $$\frac{\Delta I}{I_{0}}\approx \kappa \cdot \Delta L\left(t\right)=\kappa \cdot \frac{L_{0}}{E}P_{0}\sin\left(\omega t\right)$$

where $\kappa ={\left.\frac{\partial I}{\partial L}\right|}_{L=L_{0}}$ denotes the intensity-to-cavity-length sensitivity. When the ultrasonic wavelength $\Lambda_{s}$ is comparable to the cavity dimension $L_{0}$, spatial non-uniformity of pressure distribution along the cavity must be considered. The effective cavity displacement becomes [42,43,44]:

(9) $$\Delta L_{\mathrm{eff}}=\frac{1}{\Lambda_{s}}\int_{0}^{L_{0}}P\left(x\right)\cdot \eta \left(x\right)dx$$

where η(x) represents the normalized intensity distribution (or pressure sensitivity profile) within the cavity.

For an extrinsic FPI, the cavity is formed between two fiber end-faces, and the acoustic pressure induces mechanical displacement of the fiber tip. The resulting cavity length change is governed by [42,43,44]:

(10) $$\Delta L\left(t\right)=\frac{p\left(t\right)\cdot A}{k_{\mathrm{fiber}}}$$

where $k_{\mathrm{fiber}}$ is the effective mechanical stiffness of the fiber, which depends on its material and geometric properties.

From the above analysis, it is evident that the sensitivity of FPI-based ultrasonic detection is primarily determined by parameters such as cavity length, end-face area, material properties, and mechanical compliance. Both FBG and FPI sensors offer unique advantages in distributed and point sensing applications, but their performance is fundamentally constrained by inherent trade-offs—such as spatial averaging in long FBGs or limited bandwidth in high-finesse FPIs.

Table 3 summarizes recent research on fiber-optic ultrasonic sensors over the past five years. In the following section, we provide a detailed discussion of two key development trends in ultrasonic sensor technology: high-sensitivity sensors and broadband sensors.

#### 2.1.2. High-Sensitivity Sensors

In 2021, significant advancements in high-sensitivity fiber-optic Fabry–Perot interferometer (FPI) sensors emerged, including Guo et al.’s MEMS-fabricated silicon cantilever structure achieving 28.75 μm/Pa sensitivity at resonance [46], Moradi et al.’s molybdenum disulfide (MoS_2_) thin-film sensor with 18.8 rad/Pa phase sensitivity and excellent thermal stability [49], and Li et al.’s MEMS-beam-supported silicon diaphragm enhanced with a Fresnel zone plate for 8.4 dB sensitivity improvement [50]. The following year witnessed further innovation with Li et al.’s chitosan-based FPI hydrophone demonstrating 52.6 mV/kPa sensitivity across 100–800 kHz [51] and Chen et al.’s 3D-printed all-polymer cavity sensor achieving 189× higher sensitivity than commercial piezoelectric transducers [52].

In 2023, Chen et al. introduced a two-photon polymerization (TPP)-fabricated microcavity sensor with 889-fold sensitivity enhancement [55], while Shao et al. developed a PDMS-filled hollow-core fiber sensor exhibiting broad 1 MHz bandwidth and exceptional stability [56]. By 2024, Wei et al. achieved ultra-low noise-equivalent pressure (26.9 μPa/Hz^1/2^) through dual-resonant-cavity design [59], and Shao et al. demonstrated an anti-resonant reflecting optical waveguide (ARROW) sensor with 5.4 MHz bandwidth and superior pressure sensitivity compared to fiber Bragg grating sensors [60]. Most recently in 2025, Lu et al. reported a two-segment non-uniform fiber Bragg grating (TNFBG) achieving 21× higher SNR (85.5 dB) than standard FBGs through controlled fracture methodology [62], highlighting the continued innovation in distributed fiber-optic ultrasonic sensing technologies.

In summary, recent advancements in high-sensitivity fiber-optic ultrasonic sensors demonstrate a clear trend toward novel materials (e.g., MoS_2_, chitosan), MEMS and nanofabrication techniques (e.g., TPP), and innovative resonant structures (e.g., FZPs, dual-cavities). These developments have achieved remarkable enhancements in sensitivity, signal-to-noise ratio, and detection limits, often surpassing conventional piezoelectric and FBG sensors. Moreover, significant progress has been made in addressing practical application requirements, including expanding bandwidth, improving temperature stability, and ensuring long-term reliability, thereby paving the way for their broader implementation in complex structural health monitoring scenarios.

#### 2.1.3. Wide-Bandwidth Ultrasonic Sensors

In 2021, significant wideband sensor advancements included Li et al.’s PDMS-based Fabry–Perot sensor with controllable cavity parameters achieving 70 dB SNR and 11.11 μPa/Hz^1/2^ detection limit [46], Westerveld et al.’s silicon photonic platform sensor featuring 1.3 mPa/Hz^1/2^ NEP across 3–30 MHz with CMOS compatibility [47], and Guan et al.’s flexible fiber laser sensor enabling multi-scale photoacoustic imaging with NEP down to 43.6 Pa [51]. The following year saw Yang et al. develop a tapered multicore fiber sensor exhibiting 57.79 dB SNR and 250 kHz-4 MHz bandwidth [53], alongside Tatel et al.’s BaTiO_3_ microsphere-integrated sensor with remarkable 0.1–45.6 MHz frequency response [54]. In 2023, Yin et al. demonstrated a suspended polymer microrod sensor with 7.6 MHz bandwidth using end-face grating technology [57]. Recent breakthroughs include Lv et al.’s microsphere-type FPI sensor achieving 300 kHz-3 MHz frequency response in solid media [58], Zhao et al.’s two-photon polymerization 3D printed waveguide sensor extending bandwidth to 20 MHz [61], and Cui et al.’s vertically coupled FSPSFBG sensor maintaining only 3–5% directional sensitivity variation at high temperatures [63]. These developments collectively highlight the trend toward miniaturization, enhanced integration, and broad bandwidths through innovative fabrication techniques and structural designs that significantly expand applicability across diverse inspection environments.

Furthermore, recent advances highlight the pursuit of miniaturization and enhanced integration, alongside high sensitivity. Researchers have leveraged novel fabrication techniques like two-photon polymerization and silicon photonics to create compact, high-performance sensors with wide bandwidths and superior stability. These developments, featuring innovative structures such as tapered fibers, microspheres, and waveguides, demonstrate a strong trend towards achieving multifunctional, robust, and array-compatible fiber-optic ultrasonic sensors, effectively expanding their applicability in diverse inspection environments.

### 2.2. Active Fiber-Optic Ultrasonic NDT

The fundamental distinction of active fiber-optic ultrasonic NDT lies not in any single “core component” but in its integrated dual functionality—where the optical fiber system simultaneously serves as both ultrasonic generator and detector. Unlike passive implementations that rely on external ultrasonic sources with fiber solely functioning as receiver, active NDT achieves complete integration through coordinated transducer-sensor pairing. The photoacoustic transducer component, composed of light-absorbing materials integrated with micro-scale structures, leverages the photoacoustic effect to convert laser pulses into ultrasonic waves via optical-to-thermal-to-mechanical energy conversion. Concurrently, specialized fiber-optic sensors (such as FBGs or FPIs) detect the resulting wavefields through optical parameter modulation. This complementary transducer-sensor architecture defines the “active” nature of the system, enabling distributed ultrasonic generation without electronic components while maintaining the fiber’s inherent detection capabilities. The photoacoustic effect itself describes the physical process where a material, upon absorbing periodically modulated light (such as pulsed or amplitude-modulated continuous-wave light), converts optical energy into thermal energy, resulting in localized temperature rise, thermal expansion, and consequent pressure wave generation that manifests as ultrasonic signals.

#### 2.2.1. Photoacoustic Materials

The performance of a FOPT is fundamentally governed by the properties of its constituent photoacoustic materials and the physics of the photoacoustic effect. The photoacoustic effect describes the process where a material absorbs pulsed optical energy, converts it into heat, and subsequently generates an ultrasonic wave through thermoelastic expansion [64,65,66,67]. The efficiency of this photoacoustic conversion, the ultrasonic frequency, and the resulting acoustic pressure are critical parameters that directly determine the sensitivity and resolution of the NDT system.

The photoacoustic conversion efficiency (η) quantifies the ratio of generated acoustic energy ($E_{aco}$) to the incident optical energy ($E_{opt}$), as expressed by Equation (11) [68]:

(11) $$\eta =\frac{E_{aco}}{E_{opt}}=\frac{E_{aco}}{A\cdot F}$$

where A is the optical absorption coefficient and F is the laser fluence. The generated acoustic pressure (P) is directly proportional to the temperature rise (ΔT) of the material, which is determined by its optical absorption, density (ρ), and specific heat capacity (C), as shown in Equation (12) [64,68]:

(12) $$\Delta T=\frac{\alpha \eta E}{\rho C}$$

**Figure 4:**
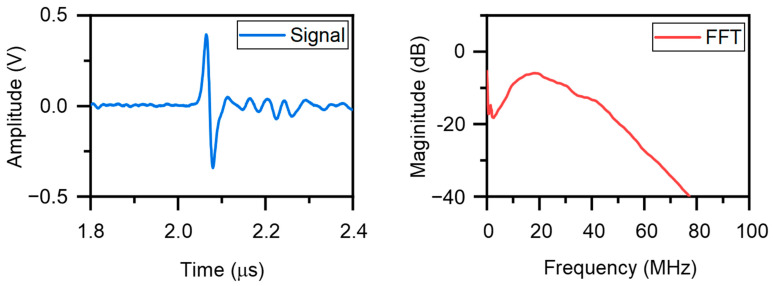
Representative photoacoustic signals and corresponding frequency spectra generated from a PDMS-graphene composite (mass ratio 10:1) and pristine PDMS under 1064 nm, 10 ns laser pulses with 1 mJ pulse energy.

Here, α is the light absorption coefficient and η is the non-radiative transition probability. The initial ultrasonic pressure can then be described by Equation (13) [64,68]:

(13) P=ΓβΔT

where β is the thermal expansion coefficient and Γ is the Grüneisen parameter, a dimensionless quantity representing the efficiency of converting thermal energy into acoustic energy. Furthermore, the center frequency ($f_{c}$) of the generated ultrasonic pulse is inversely related to the characteristic dimension (d) of the heated region and the speed of sound (c) in the material, often approximated by $f_{c}\approx c/2d$ [64]. The photoacoustic pressure generation efficiency depends critically on four material properties (Equations (11)–(13)): (1) a high optical absorption coefficient (α) ensures that most incident light is converted into heat; (2) a low specific heat capacity (C) allows for a larger temperature rise (ΔT) for a given energy input; (3) a high thermal expansion coefficient (β) enhances the volumetric strain response to heating; and (4) a large Grüneisen parameter (Γ) promotes efficient conversion of thermal energy into acoustic pressure. This explains why materials like carbon nanotubes and MXenes—despite moderate absorption—are effective when embedded in PDMS: they provide strong localized heating, while PDMS’s high β and Γ amplify the resulting acoustic output. These formulas underscore the importance of photoacoustic materials possessing a high optical absorption coefficient, large thermal expansion coefficient, low specific heat capacity, and optimized geometry to achieve high conversion efficiency, high acoustic pressure, and broad bandwidth. Notably, while PDMS exhibits relatively low intrinsic optical absorption, it possesses a high thermal expansion coefficient (~3.1 × 10^−4^ K^−1^) and excellent viscoelastic properties, making it an ideal matrix for translating photothermal energy into mechanical expansion. When combined with high-absorption nanomaterials (e.g., CNTs, CSNPs, MXenes), PDMS efficiently converts localized heating into strong thermoelastic stress, thereby enhancing photoacoustic signal generation. This functional synergy explains its widespread use in DFP-NDT transducers. Figure 4 shows an illustrative example of the ultrasonic signals excited by polydimethylsiloxane (PDMS) and graphene and their spectra. The graphene-enhanced composite exhibits higher peak amplitude and broader bandwidth compared to pure PDMS, illustrating the improved photothermal conversion efficiency enabled by carbon-based nanofillers.

In 2015, Chang et al. proposed a candle-soot nanoparticle-polydimethylsiloxane (CSNPs-PDMS) composite film as a photoacoustic transducer material, leveraging its high light absorption and rapid heat diffusion for efficient photoacoustic conversion [69]. The device generated broadband ultrasonic pulses with a center frequency of approximately 12 MHz, achieving an energy conversion efficiency of 0.441 × 10^−2^ and a peak acoustic pressure exceeding 1 MPa.

In 2018, Chen et al. introduced a photoacoustic transducer based on a multilayered carbon nanotube (CNT) yarn-gold nanoparticle-PDMS composite structure. By algorithmically optimizing the light absorption and thermoelastic expansion models, they achieved efficient acoustic field modulation [70]. This device produced broadband ultrasonic pulses centered at approximately 11.8 MHz, with an energy conversion efficiency of 2.74 × 10^−2^ and a peak positive pressure as high as 33.6 MPa.

In 2021, Li et al. developed an aligned CNT array-PDMS composite photoacoustic transducer. Their algorithmic model revealed an inverse relationship between device thickness and operating frequency, confirming its operation in thickness mode [71]. By exploiting the high anisotropic thermal conductivity of CNTs and the high thermal expansion coefficient of PDMS, the device achieved efficient photothermal-acoustic conversion. Experimental results demonstrated that a 18 μm-thick composite layer generated ultrasonic signals at ~20 MHz, with a photoacoustic conversion efficiency of ~0.251 × 10^−2^ and a peak pressure approaching 9 MPa under 10 mJ laser excitation.

Also in 2021, Du et al. presented a photoacoustic transducer based on a methylammonium lead iodide (MAPbI_3_) perovskite/PDMS composite structure [72]. Through algorithmic modeling, they elucidated the coupling mechanisms among light absorption, heat conduction, and acoustic field generation. The material’s high absorption coefficient, low specific heat capacity, and low thermal diffusivity enabled efficient photothermal-acoustic conversion. Under 532 nm laser excitation, the transducer generated broadband pulses at ~29.2 MHz, achieving a conversion efficiency of 2.97 × 10^−2^ and a peak pressure of 24.9 MPa.

In 2023, Wu et al. proposed a Ti_3_C_2_T_x_ MXene/PDMS bilayer photoacoustic transducer, establishing a multiphysics algorithmic model that coupled optical, thermal, mechanical, and acoustic fields to optimize the layer thickness–acoustic pressure relationship [73]. The device leveraged MXene’s high light absorption and near-100% photothermal conversion efficiency, combined with PDMS’s high thermal expansion. Under 532 nm laser excitation, the transducer produced ~8.06 MHz broadband pulses with a conversion efficiency of 1.25 × 10^−2^ and a peak pressure of 15.7 MPa.

In 2024, Du et al. developed a mid-infrared photoacoustic transducer using a PDMS film, with their algorithmic model analyzing the coupling between C–H bond vibrational excitation and acoustic field generation [74]. The high C–H bond density and favorable thermal expansion of PDMS enabled efficient vibration-driven photoacoustic conversion. A 25 μm thick PDMS film under 3.38 μm laser excitation generated ~10 MHz broadband ultrasonic signals, with a conversion efficiency 37.5-fold higher than conventional CNT-PDMS structures.

In 2025, Zhang et al. reported a laser-driven photoacoustic transducer based on a Ti_3_C_2_T_x_ MXene/PDMS composite film [75]. Through multiphysics field modeling, they analyzed the coupling of light absorption, heat conduction, and acoustic generation, while optimizing the film thickness–pressure relationship. Experimental results showed that a 1.2 μm thick MXene film under 532 nm laser excitation generated broadband pulses in the 7.7–8.4 MHz range, with a high conversion efficiency of 1.21 × 10^−2^ and a peak pressure of 25.3 MPa.

Critical comparison among carbon nanotubes (CNTs), perovskites (e.g., MAPbI_3_), and MXenes reveals distinct efficiency-bandwidth trade-offs. CNT-based composites offer broadband operation (up to ~20 MHz) and moderate conversion efficiency (~2.7 × 10^−2^), but suffer from dispersion instability and moderate photothermal yield. Perovskite materials exhibit higher peak efficiencies (~3 × 10^−2^) and >29 MHz bandwidths due to strong light-matter interaction, though their long-term stability under ambient conditions remains a concern. MXene-based transducers balance efficiency (~1.2–1.25 × 10^−2^), robustness, and dual functionality (e.g., corrosion protection), but operate at lower frequencies (<10 MHz), making them more suitable for large-area, low-frequency inspection. For high-frequency applications (>20 MHz), materials with high absorption and rapid thermal relaxation (e.g., perovskites, graphene) are preferred; for deep-penetration or structural monitoring, lower-frequency, thermally robust composites (e.g., MXene-PDMS) offer practical advantages.

Table 4 summarizes the key metrics, including center frequency and conversion efficiency, of various photoacoustic materials developed in recent years. Correspondingly, material selection has evolved to significantly enhance performance. Early composites like CSNPs-PDMS established a solid foundation, while subsequent innovations, such as CNT-gold nanoparticle hybrids and perovskite materials, have pushed boundaries by achieving higher center frequencies (>20 MHz) and remarkably improved efficiencies (up to ~3 × 10^−2^). The latest explorations of MXene composites and vibration-driven mechanisms continue this trend, delivering multi-megahertz signals with high acoustic pressures. This progressive development of high-performance materials is fundamental for advancing the capabilities of fiber-optic photoacoustic transducers.

#### 2.2.2. Microstructures of Single Point FOPT

**Figure 5:**
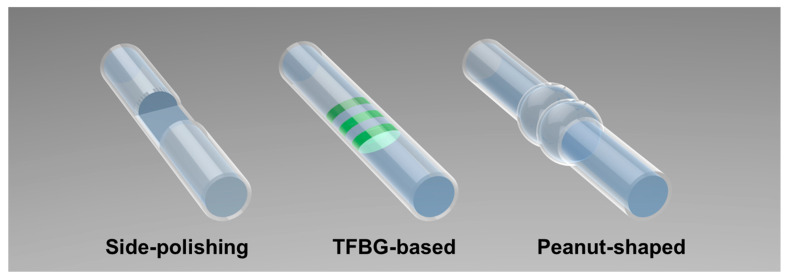
Schematic diagram of side-polishing, TFBG-based, and peanut-shaped Microstructures.

The fundamental performance of a FOPT, including its efficiency, bandwidth, and directional characteristics, is not merely determined by the photoacoustic materials. It is critically dependent on the microstructure designed at the fiber tip or along its sidewall. These intricate microstructures govern the spatial distribution of optical energy, the thermo-mechanical conversion process, and the effective coupling of generated ultrasound into the target medium. In recent years, a multitude of innovative microstructural designs have been proposed (Figure 5) to address a wide range of application requirements, from focused high-frequency imaging to large-area structural inspection. This section provides a comprehensive review of these representative microstructures in single point FOPT, detailing their fabrication methods and their impact on ultrasonic generation performance.

In 2009, Luna Innovations Incorporated pioneered a side-polishing technique to couple pulsed laser light from the fiber core into the cladding, followed by coating the polished region with a graphite-epoxy composite as both the optical absorber and thermoelastic medium, thereby fabricating an array of five side-excited FOPTs [76].

In 2020, Wang et al. developed a FOPT capable of operating at elevated temperatures up to 600 °C [77]. The device used a gold-coated multimode fiber for ultrasonic generation and integrated both a FPI and a FBG within a second fiber for detection. By bonding both fibers to a metal pipe (1.5 cm long, 4.14 cm in diameter), the system successfully detected two 0.5 mm-wide cracks.

In 2023, Li et al. proposed a tilted fiber Bragg grating (TFBG)-based laterally emitting and receiving integrated ultrasonic transducer, achieving, for the first time, simultaneous ultrasonic generation and reception from the side of a single fiber [78]. Functionalized carbon nanotube composite was deposited on the TFBG cladding to enable pulse excitation with a center frequency of 5 MHz and a 7 MHz bandwidth, yielding a SNR of 51 dB. For signal reception, intensity modulation of the core mode in the TFBG was demodulated.

In 2024, Luo et al. introduced a multi-point excitation laser-ultrasonic transducer based on a long-period fiber grating (LPFG), where precise coupling between the core and cladding modes was achieved by tuning the LPFG parameters (grating length and modulation depth) [79]. Experimentally, two excitation sources produced ultrasonic signals with peak-to-peak amplitudes of approximately 500 mV at 3 MHz, exhibiting balanced energy distribution. Also in 2024, Wu et al. proposed a peanut-shaped FOPT for NDT of large-area aluminum plates [28]. The transducer generated Lamb wave signals at 0.08 MHz and 0.42 MHz, achieving an inspection coverage of up to 50 cm.

In 2025, Kang et al. presented a FOPT based on the thermal cavitation effect [80]. By delivering low-power continuous-wave laser light through an optical fiber to heat a highly absorbent copper salt solution in a micro-encapsulated cavity, periodic bubble nucleation and collapse were induced, generating high-frequency, broadband ultrasonic pulses. Experiments demonstrated a sound pressure of 330 kPa, a repetition rate of 4 kHz, and a bandwidth of 5–17 MHz (−10 dB) under a laser power of 52 mW. In the same year, Luo et al. reported a FOPT based on collapsed photonic crystal fiber (CPCF) [81]. By precisely controlling the CPCF length, highly efficient light–heat–sound energy conversion was achieved. A 3-point excitation configuration was constructed, yielding a balanced peak-to-peak output of 1.5 V on an aluminum plate. Also in 2025, Wu et al. developed a low-frequency photoacoustic fiber transducer array filled with polyethylene (PE) particles for large-area, high-precision NDT [82]. By lowering the center frequency from 1 MHz to 0.3 MHz, the inspection area was significantly expanded to 90 × 54 cm^2^, with a localization error within 0.894 cm. Compared to the unmodified design, the SNR improved by 7 dB, the attenuation coefficient decreased from 0.262 to 0.248, and the relative bandwidth increased from 138% to 209%. Combined with pulse compression technology, the resolution improved from 0.45 cm to 0.24 cm. In a thick plate of 30 × 30 × 1.5 cm^3^, a localization error of 0.781 cm was achieved, demonstrating robustness against multimode interference. Also in 2025, Li et al. proposed an MXene-PDMS composite anti-corrosion coating that functions as a photoacoustic transducer array by embedding fiber sensors and leveraging the high photothermal conversion efficiency of MXene (~90%) [83]. The coating simultaneously excited multi-point Lamb waves over a 90 × 54 cm^2^ area, enabling precise localization of a 0.5 mm defect with an error of only 0.894 cm and a positioning accuracy of 97.4%.

Table 5 summarizes recent methods and photoacoustic materials employed in single point FOPTs. In recent years, significant progress has been made in the development of FOPTs, as exemplified by the diverse range of structures and mechanisms presented. Building on the foundational side-polishing technique, researchers have innovated with advanced functional materials—such as MXene-PDMS for efficient energy conversion—and engineered structures like TFBGs, LPFGs, and CPCFs to achieve multi-point excitation and side-wall integration. Novel physical mechanisms, including thermo-cavitation, have been explored to generate broadband ultrasonic. These advancements have collectively enabled the creation of transducers with enhanced functionalities, such as operation in extreme environments, large-area coverage, and integrated sensing capabilities, thereby significantly advancing the field toward practical, high-performance distributed photoacoustic NDT systems.

## 3. Distributed Fiber-Optic Photoacoustic Transducer Array (DFOPTA) Method

**Figure 6:**
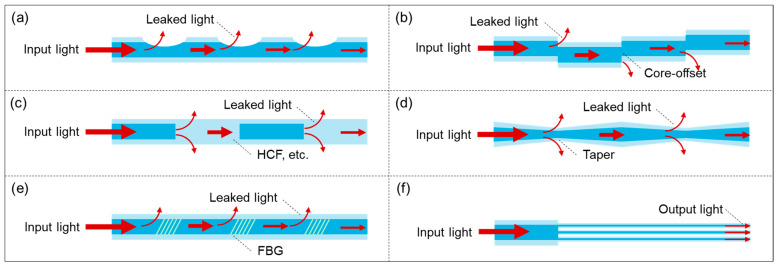
Schematic structures of six DFOPTAs. (**a**) Side wall leakage; (**b**) Core-offset; (**c**) Splicing of different fibers; (**d**) Tapering or collapsing; (**e**) Grating structures; (**f**) Multi-channel.

Distributed fiber-optic photoacoustic transducer arraying refers to methods that transform a single-point FOPT structure into a multi-point array. By arraying, a one-dimensional point sound source FOPT can be converted into a two-dimensional ultrasonic array DFOPTA, greatly expanding its application scenarios. Based on current research progress, there are six primary arraying techniques: side wall leakage, core-offset, splicing of different fibers, tapering or collapsing, grating structures, and multi-channel, as illustrated in Figure 6. The research progress for each structure is discussed in detail below.

### 3.1. Side Wall Leakage

The side wall leakage strategy involves polishing the cladding of an optical fiber to allow light to escape radially from the core into an external photoacoustic absorber. As illustrated in Figure 6a, this geometric modification reduces the cladding thickness, weakening optical confinement and enabling evanescent or direct radiative coupling of guided light into the surrounding functional coating—where it is absorbed to generate ultrasound.

In 2009, Luna Innovations Incorporated pioneered a method by polishing the side-walls of an optical fiber to couple pulsed laser light from the core into the cladding [76]. The polished sections were then coated with a graphite-epoxy composite, serving as both the light-absorbing and thermal expansion material. This approach enabled the fabrication of a 5-point side-emitting DFOPTA. The array’s detection area was 32 cm × 6 cm, exciting 1 MHz ultrasonic waves with an amplitude of 320 mV on a 33 cm × 30 cm aluminum thin plate.

In 2018, Wang et al. proposed a multi-point, distributed all-optical photoacoustic sensing system for monitoring rebar corrosion [84]. The system used a gold nanoparticle composite as the PA source, combined with a 532 nm nanosecond pulsed laser and a 400 μm multimode fiber, to generate 8 MHz ultrasonic pulses at a spatial resolution of 0.4 mm. By detecting the acoustic spectral changes in rebar sections at different corrosion levels with four FBG arrays, they found that a 0.175 MHz decrease in the center frequency corresponded to a mass loss of 0.02 g, following an exponential relationship, with a localization error controlled within 0.781–0.894 cm.

### 3.2. Core-Offset

The core-offset method fabricates an asymmetric splice between two fibers, redirecting light to one side of the fiber, enabling localized and controllable excitation where a photoacoustic material is applied. As shown in Figure 6b, the lateral misalignment induces modal mismatch and scattering at the fusion point, promoting asymmetric power distribution and side-coupling into the cladding and coating region.

In 2017, Dong et al. introduced a multi-point, distributed laser-ultrasonic generation technique based on a fiber core-offset structure, achieving balanced multi-point excitation along the fiber axis [85]. By precisely controlling the offset distance, they managed the modal coupling ratio and constructed a 5-point excitation system. It produced balanced ultrasonic signals on an aluminum plate (peak-to-peak voltage: 482–526 mV) with a low localization error of 0.781 cm. The system employed a 1550 nm, 5 ns pulse width, 3 kHz repetition rate pulsed laser, coupled with a high-power EDFA amplifier and a graphite/epoxy composite absorber, to achieve broadband excitation below 20 MHz. Chemical etching reduced the cladding diameter to 32 μm, increasing the signal amplitude to 210 mV.

**Figure 7:**
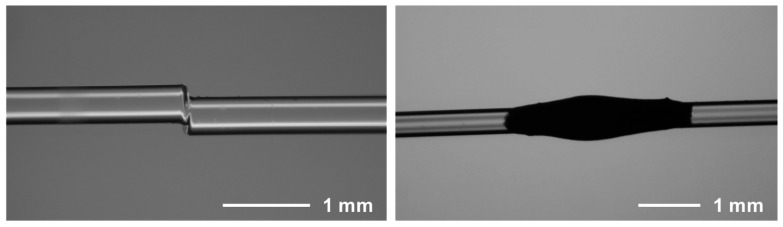
Micrograph of a core-offset transducer prepared using the method of Li et al. [83].

In 2025, Wu et al. proposed a 6-point DFOPTAs based on a core-offset structure [82]. The system enabled multi-point synchronous ultrasonic excitation over a 90 × 54 cm^2^ area. By detecting 0.5 mm flaws using Lamb waves, it achieved a localization error of only 0.894 cm with a positioning accuracy of 97.4%. Also in 2025, Li et al. developed an MXene-PDMS composite coating that serves dual functions of corrosion protection and structural health monitoring. By embedding a core-offset structure (Figure 7) into this coating, they achieved 4-point DFOPTAs [83].

### 3.3. Splicing of Different Fiber

The splicing of different fibers technique leverages dissimilar fiber properties (e.g., core/clad diameter, refractive index) at a fusion splice to induce controlled light scattering into the surrounding PA material. As depicted in Figure 6c, the mode field mismatch between dissimilar fibers (e.g., SMF and HCF) causes partial reflection and radiation of light at the joint, enabling defined side-emission zones.

In 2019, Li et al. proposed a multi-point laser-ultrasonic excitation structure based on a HCF concept by splicing a single-mode fiber (SMF) with different lengths of HCF [86]. This design enabled controllable coupling of laser energy to the side of the fiber. A 5-point excitation system was built, achieving coupling efficiencies from 20.17% to 88.10% for HCF lengths ranging from 90 μm to 300 μm, generating Lamb wave signals with a peak-to-peak voltage of approximately 510 mV, a center frequency of 4 MHz, and a bandwidth of about 10 MHz, with a localization error as low as 0.781 cm. The system used a 1550 nm, 5 ns pulse, 3 kHz repetition rate pulsed laser, in combination with a graphite-epoxy absorber and cladding etching technology, to enhance both energy utilization and signal consistency.

In 2024, Zhou et al. proposed a multi-point, energy-balanced laser-ultrasonic transducer based on a single-mode-multimode-thin-cladding (SMTC) fiber structure [87]. By tuning the length of the multimode section, they precisely controlled the coupling efficiency (33.2%, 50.4%, 89.8%), achieving three balanced excitation points on a 100 × 100 × 1 mm^3^ aluminum plate. A CSNP–PDMS composite served as the PA source, yielding a peak-to-peak signal of 1.65 V, improving the energy conversion efficiency to 413.7 mV/J—nearly 83% higher than the graphite–epoxy resin. The frequency bandwidth reached 11.71 MHz (center frequency ~3.3 MHz) with excellent signal stability (standard error < 0.0065 over 8 h).

The following year, Zhou et al. proposed another multi-point transducer based on a single-mode-multimode-single-mode (SMS) fiber structure [88], again precisely controlling the coupling ratio by adjusting the multimode segment length. A 4-point energy-balanced excitation system was constructed. For distributed application validation, all four excitation points achieved a peak-to-peak output of approximately 1018 mV with a center frequency close to 4 MHz, demonstrating good signal consistency.

### 3.4. Tapering or Collapsing

**Figure 8:**
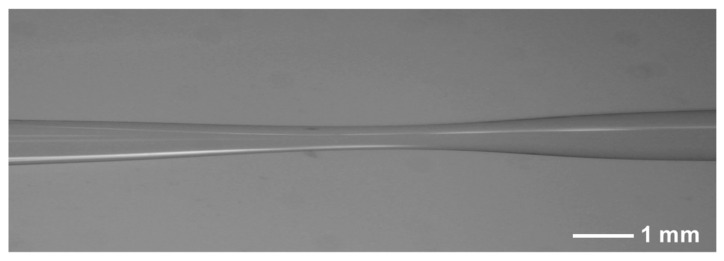
Micrograph of a tapering microstructure [83].

The tapering or collapsing method involves thermally tapering or laser-collapsing a section of fiber to modify its waveguide properties, creating localized regions where light is forced out. As seen in Figure 6d, the reduced diameter increases the evanescent field and alters mode propagation, leading to enhanced light leakage into the surrounding medium for efficient photoacoustic conversion. Micrograph of a tapering microstructure is shown in Figure 8.

In 2017, Tian et al. proposed a multi-point DFOPTAs based on an array of core-opened tapers [89]. By adjusting the taper length, they controlled the coupling of light from the core to the outer cladding. A 4-point excitation system was built, with coupling ratios of 24.14%, 33.01%, 49.51%, and 87.8%, achieving balanced ultrasonic signal strength (~0.01 mJ laser energy per point, peak-to-peak output: 450–543 mV).

In 2025, Luo et al. proposed an all-fiber laser-ultrasonic transmitter based on a CPCF [81]. By precisely controlling the length of the CPCF section, they tailored the light-coupling coefficient, constructing a three-section PCF–CPCF–PCF microstructure. This enabled 3-point ultrasonic excitation on an aluminum plate, with a balanced peak-to-peak output of ~1.5 V for each point.

### 3.5. Grating Structures

This class of multiplexing utilizes periodic refractive index variations in the fiber core to reflect light at specific wavelengths, enabling wavelength-division multiplexed (WDM) excitation. As illustrated in Figure 6e, grating structures (e.g., TFBG, LPFG) satisfy the Bragg condition to back-reflect or couple core light into cladding modes at precise locations—acting as built-in, wavelength-selective taps for distributed excitation.

In 2013, Tian et al. proposed a novel method for distributed laser-ultrasonic generation using asymmetric TFBGs through their cladding (or “ghost”) modes [90]. By designing a TFBG array with different ghost-mode wavelengths and coating their tips with a high-absorption material, they achieved efficient out-coupling of specific-wavelength lasers at designated locations. In experiments, three TFBGs resonated with 1544.1 nm, 1548.9 nm, and 1555.2 nm lasers, successfully generating ultrasonic pulses (3 kHz repetition rate, >8 MHz bandwidth, ~0.33 mJ pulse energy) at multiple points.

In 2025, Luo et al. introduced a multi-point laser-ultrasonic sensor based on LPFG [79]. By tuning the grating length and modulation depth, they precisely controlled the coupling ratio between the core mode and cladding modes, with experimental coupling efficiencies ranging from 52.31% to 95.27%. Under 3 kHz repetition rate, 5 ns-pulse excitation, dual ultrasonic sources generated stable signals with a peak-to-peak voltage of ~500 mV, a center frequency of ~3 MHz, and a bandwidth of 2–4 MHz.

### 3.6. Multi-Channel

The multi-channel approach uses a bundle of independent optical fibers, each functioning as a separate excitation channel, offering the highest design freedom and signal isolation. As shown in Figure 6f, this architecture avoids in-fiber power splitting entirely, delivering full pulse energy directly to discrete transducer sites with minimal crosstalk.

In 2025, Feng et al. proposed an embeddable, multi-channel all-optical-fiber acousto-ultrasonic system, enabling multi-point laser excitation in carbon fiber reinforced polymers (CFRP) coupled with detection via an FBG array [91]. The system used a 1064 nm pulsed laser (2 W) and a 250 μm double-clad fiber for excitation, combined with a 5 mW continuous-wave laser and a 5 mm FBG array for detection. It exhibited strong directionality, high signal-to-noise ratio, and immunity to electromagnetic interference. Experiments demonstrated the stable generation of ultrasonic waves with a center frequency of ~1.1 MHz and a duration of 12–18 μs. No signal degradation was observed after 3.6 million pulse cycles under continuous monitoring. In a drilling damage test, the four-channel signals attenuated by 70%, 97%, 99%, and 60%, respectively, successfully identifying the damage location.

**Figure 9:**
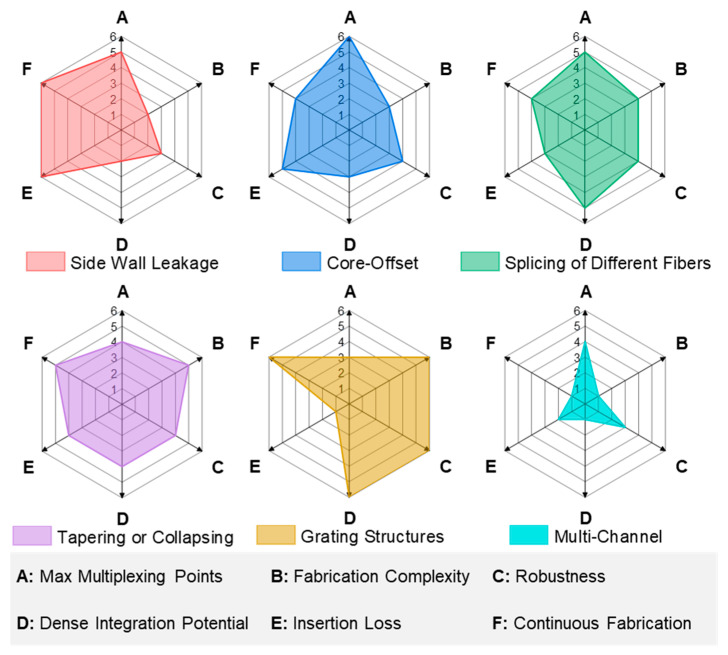
Performance comparison of six DFOPTA methods across six critical metrics.

Table 6 summarizes the recent research on distributed FOPT arraying methods. To enable distributed and multi-point ultrasonic excitation, various array designs for FOPTs have been developed. Six primary methods have emerged: side wall leakage, core-offset, splicing of different fibers, tapering or collapsing, FBG structures, and multi-channel systems. These techniques enhance spatial coverage and enable balanced signal generation across multiple points, crucial for large-area structural health monitoring. By leveraging advanced micro-structuring and novel photoacoustic composites, these configurations form integrated systems that act as functional sensor networks, paving the way for sophisticated, deployable NDT solutions with high spatial resolution and multi-point damage localization capabilities. A comprehensive comparative assessment of these methods—spanning multiplexing capacity, fabrication complexity, robustness, integration potential, insertion loss, and scalability toward continuous fabrication—is presented in Figure 9. This radar chart provides a visual synthesis to guide technology selection based on specific application requirements, such as industrial deployability or high-density sensing.

## 4. Imaging Algorithms Applied to DPF-NDT

Effective data interpretation is paramount to realizing the full potential of DPF-NDT, with advanced ultrasonic imaging algorithms serving as the key to transforming raw multi-point signals into intuitive, high-resolution damage visualizations. The predominant algorithmic approaches can be broadly categorized into the total focusing method (TFM), the reconstruction algorithm for probabilistic inspection of damage (RAPID), and artificial intelligence (AI)-based techniques. Critically, the imaging algorithms developed for both conventional piezoelectric and laser ultrasonics provide a valuable foundation and offer significant guiding principles for their adaptation and optimization within the unique domain of fiber-optic photoacoustics. Table 7 summarizes recent research achievements in ultrasonic imaging algorithms, and the subsequent sections provide a detailed discussion of each of these prominent methods.

While a variety of imaging algorithms—including TFM, RAPID, and AI-based techniques—have been applied to DFP-NDT, it is critical to recognize that most are adaptations of conventional ultrasonic imaging frameworks originally developed for piezoelectric or laser-ultrasonic systems. These legacy algorithms often operate under assumptions incompatible with the unique characteristics of DFP-NDT: sequential single-source excitation, uniform transducer spacing, and synchronized signal acquisition. In contrast, DFP-NDT systems exhibit multi-source synchronous excitation, high channel count, and distributed multi-point excitation, which introduce significant complexity in signal coherence, interference modeling, and data fusion. Without algorithmic redesign, these features can lead to artifacts, mislocalization, and reduced imaging fidelity. Therefore, the transition from algorithmic adaptation to native algorithm development is essential for unlocking the full potential of distributed photoacoustic arrays.

### 4.1. Total Focusing Method

In 2019, Xu et al. proposed a defect localization strategy combining matching pursuit with time-reversal focusing [92]. The MP algorithm utilized a custom overcomplete dictionary to achieve sparse decomposition through time-shift and amplitude modulation, demonstrating strong noise suppression and low SNR adaptability. The time-reversal approach leveraged spatiotemporal focusing to concentrate energy within a finite element reference model. This integration achieved axial localization errors of only 0.4–1.2% for pipeline defects.

In 2022, Xu et al. proposed a waveform correlation factor-weighted TFM for Lamb wave array damage imaging [95]. Building on conventional TFM amplitude imaging, this approach introduced waveform correlation coefficients as weighting factors to integrate signal amplitude and waveform similarity, effectively suppressing background noise. Notably, it required no baseline data and supported linear/circular arrays. Experiments demonstrated ~20 dB noise reduction versus conventional TFM, with positioning errors ≤ 8 mm (mean: 5.46 mm; standard deviation: 1.39 mm).

Also in 2022, Fan et al. evaluated time-reversal imaging with multiple signal classification (TR-MUSIC) and phase-coherent imaging with multiple signal classification (PC-MUSIC) super-resolution imaging algorithms for metallic defect localization [96]. Using full matrix capture data, singular value decomposition separated signal/noise subspaces for point-like and extended defect imaging. Key features included diffraction-limit breakthrough, with PC-MUSIC enhancing length/angle characterization of extended defects via phase consistency. The result of this algorithm, point-like defect errors < 8%, extended defect length measurement errors as low as 9.62%, and angular errors of 13.3%.

In 2023, Cui et al. introduced a baseline-free Lamb wave damage localization algorithm based on compressed sensing [97]. The method extracted scattered waves via frequency-wavenumber domain filtering and applied short-term average/long-term average (STA/LTA) time-delay extraction for probabilistic imaging. Its core innovation used compressed sensing to reconstruct sparse wavefields, achieving 86.7% data compression with only 10 sampling points.

**Figure 10:**
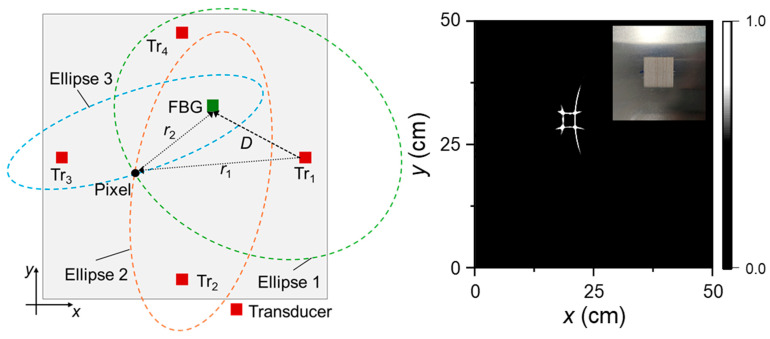
Adapt from the results of the ellipse TFM imaging algorithms in an all-fiber photoacoustic system [28].

Also in 2023, Wu et al. applied the Time-Varying Filter Empirical Mode Decomposition (TVF-EMD) method to decompose complex ultrasonic signals, resolving the mode mixing problem caused by acoustic impedance mismatch [28]. Integration with group velocity calculations enabled successful identification of different Lamb wave modes. Subsequently, the authors proposed an elliptical trajectory method for crack localization and imaging and developed a Tukey window filter to eliminate direct waves and boundary reflections, thereby significantly improving the accuracy and resolution of crack visualization in ultrasonic non-destructive testing (Figure 10).

In 2024, Li et al. proposed a path amplitude-matched baseline-free Lamb wave defect localization algorithm [98]. By comparing direct wave amplitudes across circular array sensor pairs and normalizing boundary reflected waves to eliminate excitation variations, it enabled baseline-free damage localization. Its distinctive feature determined defect-proximate paths via amplitude differentials and identified defect zones through multi-path convergence. With the application of this method, 13 mm localization bias with 16 sensors, reduced to 0.42 mm with 32 sensors.

### 4.2. Reconstruction Algorithm for Probabilistic Inspection of Damage

In 2022, Teng et al. developed an enhanced RAPID for damage localization in high-speed train crossbeams [94]. The method combined correlation-based damage indices with time-delay difference correction probability distribution functions to distinguish “through-damage” from “adjacent-damage” propagation paths, enabling fused imaging. Key innovations included improved localization accuracy and elimination of traditional RAPID blind spots. Experimental results showed absolute positioning errors < 8 mm (mean: 5.46 mm; standard deviation: 1.39 mm).

**Figure 11:**
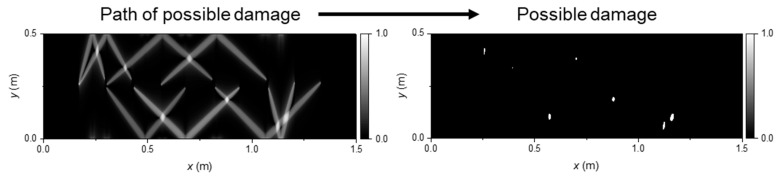
Adapt from the results of the RAPID imaging algorithms using hybrid Copula functions [100].

In 2025, Fu et al. presented an improved RAPID algorithm using hybrid Copula functions [100]. A bivariate hybrid Copula model precisely characterized nonlinear correlations under multi-source excitation, integrating correlation and energy indices into a baseline-free damage index. This innovation eliminated environmental interference effects, enabling large-scale lightweight inspection. Validation on a 2000 mm × 1000 mm × 0.5 mm aluminum plate showed average absolute errors of only 3.83 mm (0.31% relative error) for coexisting damages in Figure 11.

Also in 2025, Luo et al. proposed a Nonlinear-Exponentially weighted RAPID (NE-RAPID) with adaptive time-reversal for delamination detection in composites [101]. Key features included replacing linear weights with exponential decay weights and introducing geometric shape correction functions to alleviate path-convergence shadowing. Validated on CFRP plates, NE-RAPID achieved positioning errors as low as 1.41 mm—significantly outperforming conventional RAPID (errors > 30 mm).

### 4.3. Artificial Intelligence-Based

In 2021, Soman et al. introduced a genetic algorithm-based actuator placement optimization method [93]. Their cost function incorporated coverage^2^ (ensuring ≥ 99.6% coverage for ≥2 actuator-sensor pairs) and coverageR (95.6% edge reflection coverage), accounting for the directional sensitivity and passive characteristics of Fiber Bragg Grating (FBG) sensors. A two-step strategy determined actuator quantity and position, achieving high coverage and precise damage localization in plate structures.

In 2024, Tunuković et al. developed an unsupervised defect detection algorithm using automatic gating and autoencoders [99]. Density-based spatial clustering enabled automatic gating to remove strong front/back wall echoes, while processed B-scans were fed into a convolutional autoencoder network for reconstruction error discrimination. Key advantages included no requirement for defective training samples, strong generalizability, and rapid inference. Validated on CFRP composites, it processed 2070 B-scans in 1.26 s with ROC-AUC up to 0.922 for simple samples.

In 2025, Hawwat et al. introduced a support vector machine-based crack classification algorithm [102]. Trained on synthetic databases from experiments and finite element simulations, it extracted frequency-domain features (e.g., center frequency) for training. Key innovations included annular focusing for circumferential crack positioning and precise geometric parameter classification via SVM. Validation showed classification accuracy > 90% in most cases, with crack length/depth errors ≤ 5 mm and ≤10% of wall thickness, respectively.

Recent advancements in ultrasonic imaging algorithms—including TFM variants, enhanced RAPID implementations, and AI-driven approaches—have substantially improved defect localization accuracy (often achieving <5 mm error) while enabling baseline-free operation and strong noise suppression in fiber-optic photoacoustic NDT systems. These methods leverage innovations like waveform correlation weighting, nonlinear exponential modeling, and deep learning to enhance reliability across diverse structural inspections. However, a critical limitation persists: current algorithms exhibit insufficient optimization for FOPT-specific scenarios involving simultaneous multi-source excitation, as most studies focus on sequential single-source configurations. This gap hinders real-time monitoring capabilities for large-area structures where synchronous excitation from distributed arrays is essential, necessitating dedicated algorithmic development for true multi-point concurrent operation.

## 5. Application of DFOPTA

The distributed fiber-optic photoacoustic transducer array (DFOPTA) has emerged as a transformative approach in structural health monitoring, offering unique capabilities for large-area, multi-point damage assessment without electromagnetic interference. By seamlessly integrating photoacoustic conversion principles with distributed optical sensing architectures, DFOPTA overcomes conventional limitations of both piezoelectric and laser-ultrasonic methods while enabling unprecedented flexibility in inspection scenarios. As evidenced by the chronological progression of research summarized in Table 8, this technology has rapidly expanded from early corrosion monitoring applications to sophisticated defect characterization across diverse material systems. The following sections systematically examine DFOPTA’s practical implementations, highlighting how its distinctive features—including customizable frequency response, spatially resolved excitation, and compatibility with harsh environments—address critical challenges in real-world non-destructive testing scenarios while delivering exceptional measurement fidelity.

### 5.1. Damage Detection in Metal Materials

In 2023, Wu et al. employed a four-point DFOPTA for NDT of a large-area aluminum plate, using the array to generate ultrasound and combining it with a modal decomposition algorithm to mitigate mode mixing caused by acoustic impedance mismatch—thereby simplifying signal interpretation. The method was primarily targeted at crack visualization and localization in structural components. Key measurement metrics included ultrasonic signal amplitude, transmission loss, and spatial resolution. The system achieved a detection coverage of 50 × 50 cm^2^ with a crack localization resolution of 1 mm [28]. Defect characterization relied on elliptical trajectory analysis of multi-position ultrasonic echoes, enabling precise crack localization and shape recognition through amplitude attenuation and waveform variation.

**Figure 12:**
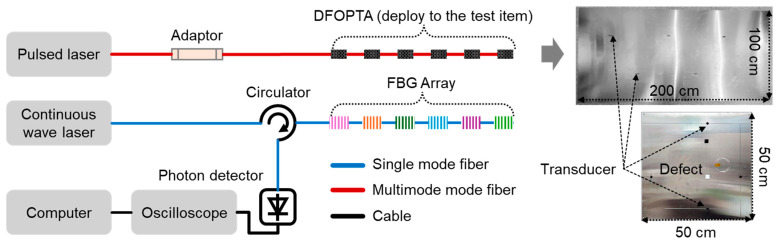
Schematic diagram of the metal damage detection system adapted from references [28,82,83].

In 2025, the same group proposed a low-frequency DFOPTA for large-area, high-precision NDT as shown in Figure 12, with a key innovation involving the integration of a functional layer of polyethylene particles into the transducer [82]. By selectively attenuating high-frequency components, the center frequency was reduced from 1 MHz to 0.3 MHz, significantly decreasing propagation loss and extending the effective detection range. This approach is particularly suited for online defect detection in composites and thick plates, addressing signal attenuation and missed detections common in conventional high-frequency methods. The system achieved a detection area of 90 × 54 cm^2^, with a localization error below 0.894 cm for a single-fiber six-element array, maintaining robust performance (≤0.781 cm) even in thick specimens under multimodal interference.

Also in 2025, Li et al. developed a DFOPTA based on an MXene (Ti_3_C_2_T_x_)-PDMS smart anti-corrosion coating, achieving dual functionality: structural protection and health monitoring [83]. The system successfully identified 0.5 mm surface cracks with a positioning accuracy of 97.4%, demonstrating high sensitivity and spatial resolution. Defect detection utilized Lamb waves generated via the photoacoustic effect, with spatial localization achieved by comparing waveform energy and time-of-flight across different sensing paths.

### 5.2. Damage Detection in Composite Materials

In 2025, Wei et al. applied a DFOPTA based on CPCF to detect damage in CFRP laminates of 2 mm and 4 mm thickness [81]. By analyzing the time- and frequency-domain responses of the DFOPTA on different thicknesses and using a piezoelectric transducer (PZT) for reception, they successfully imaged defects at varying depths, demonstrating the method’s capability in multilayer composite inspection.

**Figure 13:**
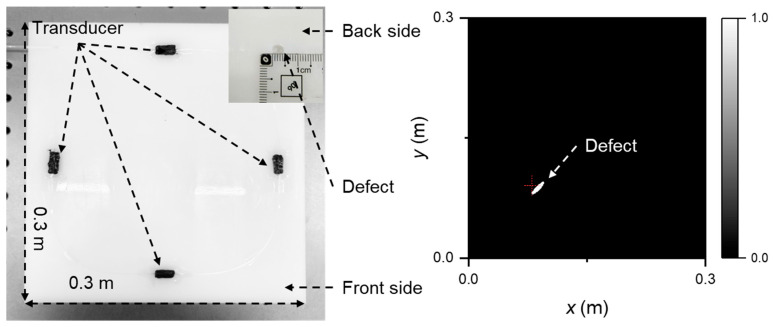
Results of composite material damage detection adaptively adjusted from reference [74].

Also in 2025, Feng et al. utilized a multi-channel DFOPTA for structural health monitoring of CFRP, leveraging pulse laser-induced optoacoustic conversion at four parallel fiber ends to generate directional ultrasonic guided waves [91]. The system employed FBG sensors combined with edge-filter demodulation for multi-channel synchronous detection. Targeted at in-service CFRP structures, key measurement parameters included wave arrival time (~12 μs), frequency (~1.1 MHz), and amplitude attenuation. Experiments showed signal attenuation of 70%, 97%, 99%, and 60% across four channels after drilling-induced damage, highlighting directional sensitivity and channel-specific response. Defect localization was based on comparative analysis of waveform energy and attenuation levels, with amplitude differences used to pinpoint damage zones. The system exhibited high directionality, embeddability, and multi-channel capability. In the same year, Wu et al. employed a low-frequency DFOPTA for damage detection in a polyethylene composite plate with a penetration depth of 1 cm (Figure 13). By arranging four transducer elements on the front surface, they successfully identified and localized a circular hole defect on the back surface with an error of only 0.781 cm [82].

### 5.3. Corrosion Detection

In 2018, Du et al. applied DFOPTA for early-stage corrosion monitoring in steel rebar, aiming to quantify mass loss and corrosion extent [84]. The method employed gold nanocomposites to generate ultrasonic pulses via the photoacoustic effect, coupled with a distributed FBG array for multi-point sensing. An algorithm based on fast Fourier transform extracted ultrasonic spectral features, establishing an exponential relationship between central frequency downshift and mass loss, enabling quantitative corrosion assessment. Experimental results confirmed a strong correlation between frequency reduction and corrosion-induced mass loss, demonstrating high-sensitivity, non-contact, and distributed corrosion detection within concrete.

The comprehensive review of DFOPTA applications presented in this section demonstrates its significant evolution from a novel sensing concept to a practical structural health monitoring solution with diverse industrial utility. Across metal structures, composite materials, and corrosion detection scenarios, DFOPTA consistently achieves remarkable performance metrics—including large-area coverage (up to 90 × 54 cm^2^), high spatial resolution (down to 1 mm), and exceptional robustness in multimodal interference environments-while operating without direct electrical connections. These capabilities stem directly from its innovative integration of photoacoustic transduction with distributed fiber-optic architectures, enabling simultaneous multi-point ultrasonic excitation with precise spatial control. Notably, the technology’s adaptability is evident in its successful application to both traditional metallic structures and advanced composite materials, with specialized configurations addressing domain-specific challenges through frequency tuning, material innovations, and algorithmic enhancements.

## 6. Challenges and Trends

Despite significant progress in DFOPTAs, several critical challenges must be addressed to enable broader industrial adoption. Simultaneously, emerging trends present promising pathways for advancement.

### 6.1. Multiplexing Number

First, insufficient multiplexing number remains a fundamental limitation. As evidenced by Table 4, current DFOPTA implementations typically achieve only 3–6 multiplexed points, substantially fewer than conventional piezoelectric arrays that often employ dozens of elements. While advanced microstructures—such as core-offset configurations achieving 5-point excitation [85] and splicing techniques enabling 5-point systems [86]—have pushed boundaries, scaling beyond 10 points without signal degradation poses significant optical and thermal challenges. The inherent trade-off between spatial resolution and system complexity prevents DFOPTAs from fully addressing large-scale structures requiring dense coverage.

Regarding multiplexing capabilities, advancing microstructure engineering promises dramatic improvements. Recent developments indicate three promising pathways: First, hybrid techniques combining side-wall leakage with core-offset methods [83,85] show potential for denser point configurations; second, novel photoacoustic materials like MXene-PDMS composites [75] with near-unity photothermal conversion efficiency could enable higher signal-to-noise ratios even at elevated multiplexing rates; third, innovations in wavelength-selective techniques—exemplified by tilted fiber Bragg grating (TFBG) arrays operating at distinct ghost-mode wavelengths [90]—offer foundation for wavelength-division multiplexing schemes. These approaches collectively suggest that breaking through the 10-point barrier is technologically feasible within the next 5 years. However, scaling toward higher channel counts is fundamentally constrained by the total optical power budget, localized heat accumulation, and inter-channel optical or acoustic crosstalk. Emerging solutions such as wavelength-division multiplexing (WDM) and time-division excitation offer pathways to mitigate these physical limitations.

### 6.2. Application Scenario

Second, limited application scenarios constrain practical adoption. Current research, as summarized in Table 6, focuses predominantly on controlled environments involving aluminum plates (2023–2025) and CFRP (2025). While studies like Du et al.’s 2018 corrosion monitoring [84] demonstrate functionality in concrete-embedded rebar, applications under extreme conditions remain scarce. The ability of DFOPTAs to operate at temperatures up to 600 °C through gold-coated multimode fibers [77] represents progress, yet comprehensive validation across diverse industrial environments—including high-radiation settings, deep-sea operations, and aerospace structures—remains incomplete. Even promising innovations like MXene-PDMS smart coatings [83] have yet to demonstrate robustness across the full range of operational complexities encountered in practice. Nevertheless, long-term deployment demands enhanced environmental durability—particularly resistance to humidity-induced swelling, UV degradation, and chemical corrosion—which remains a critical barrier for field applications.

For expanding application scenarios, multi-functional integration represents a clear trajectory. The MXene-PDMS composite coatings developed by Li et al. [83], which merge corrosion protection with structural health monitoring capabilities, exemplify a critical shift toward systems that provide value beyond mere defect detection. Similarly, the low-frequency DFOPTA for large-area, high-precision NDT based on polyethylene particle functional layers [82] demonstrates targeted adaptations for specific material challenges. Emerging directions include enhanced environmental resilience through novel encapsulation strategies, optimization for non-planar surfaces through flexible coating technologies, and development of materials tailored for specific wavelength sensitivities to address domain-specific requirements.

### 6.3. Algorithm Adaptability

Third, low algorithm adaptability creates a significant bottleneck in maximizing DFOPTA capabilities. As previously noted, most existing ultrasonic imaging algorithms—including TFM variants, RAPID, and AI-based approaches—are primarily designed for sequential, single-source ultrasonic excitation rather than DFOPTA’s distributed, multi-point architecture. This mismatch fundamentally limits the exploitation of DFOPTAs’ unique advantages: simultaneous multi-point excitation and synchronous detection. Studies such as those employing matching pursuit with time-reversal focusing [92] demonstrate only moderate improvements for distributed systems. Furthermore, algorithms optimized for traditional metallic structures struggle with composite materials’ complex anisotropy and multi-layered architectures, as highlighted in CFRP applications [91]. Consequently, while DFOPTAs provide rich distributed data, current algorithms cannot fully leverage this potential for real-time, high-fidelity imaging across diverse structural configurations.

In algorithm development, DFOPTA-specific computational frameworks are rapidly emerging as a research priority. Recent efforts reveal three promising directions: First, machine learning approaches that leverage distributed data characteristics—such as unsupervised algorithms using automatic gating and autoencoders presented by Tunuković et al. [99]—are beginning to address the unique noise profiles of DFOPTA systems; second, physics-informed neural networks that incorporate known optical-thermal-acoustic conversion relationships—similar to the multiphysics models described for MXene/PDMS transducers [51]—promise significant efficiency gains; third, algorithms specifically designed for simultaneous multi-source excitation, like Fu et al.’s hybrid Copula function-enhanced RAPID [101], represent important steps toward optimizing for DFOPTA’s distinctive architecture. Crucially, future algorithmic development will require closer integration with DFOPTA-specific signal characteristics rather than adaptation of conventional approaches.

These converging trends indicate that DFOPTA technology is poised to transition from laboratory demonstrations to practical industrial applications within the next decade. Realizing this potential requires collaborative efforts across materials science, photonics engineering, and computational imaging to address the remaining challenges while capitalizing on emerging opportunities. The integration of advanced multiplexing techniques, domain-specific application tailoring, and purpose-built algorithms will ultimately enable DFOPTAs to fulfill their promise as comprehensive structural integrity monitoring systems for complex engineering infrastructures.

## Figures and Tables

**Table 1:** Comparative analysis of ultrasonic NDT technologies.

Feature	Piezoelectric UT	Laser Ultrasonics	DFP-NDT
Excitation mechanism	Electrical actuation	Free-space laser irradiation	Fiber-optic photoacoustic excitation
Cabling requirement	High (per transducer)	Low (laser source)	None (all-optical)
EMI immunity	Poor	Good	Good
Spatial coverage	Limited by wiring	Limited by beam size/scanning	Distributed, embeddable
Deployment flexibility	Low (rigid mounting)	Medium (optical access needed)	High (conformal coating possible)
Real-time multipoint actuation	Possible (complex)	Possible (scanned)	Emerging (limited by multiplexing)

**Table 2:** Architectural comparison between passive and active fiber-optic ultrasonic systems.

Feature	Passive Fiber-Optic Ultrasonic Method	Active Fiber-Optic Ultrasonic Method
Excitation source	External (PZT, laser, acoustic emission)	Fiber optical transducer
Sensing mechanism	Optical demodulation of strain/pressure	Same
System architecture	Sensor-only	Transducer and sensor
Spatial coverage	Discrete points or limited arrays	Distributed, embeddable
Power delivery	Electrical cables or laser for exciter	All-optical

**Table 3:** Research on fiber-optic ultrasound sensors in the past five years.

Year	Method	Features	Sensitivity	Bandwidth (MHz)	Reference
2021	FPI	Multiple transfer	-	≤0.43	[45]
2021	FPI	Silicon cantilever	28.75 μm/Pa	-	[46]
2021	Silicon photonics	Optomechanical waveguide	-	3.0–30.0	[47]
2021	FPI	Bending fiber	-	18.0	[48]
2021	FPI	molybdenum disulfide (MoS_2_) diaphragm	18.8 rad/Pa	0.008–0.01	[49]
2021	FPI	beam-supported structure	−12.4 dB re. 1 V/Pa	-	[50]
2022	FPI	Chitosan diaphragm	52.6 mV/kPa	0.1–0.8 MHz	[51]
2022	FPI	All-polymer cavity	189 times that of the commercial sensor	0.018–0.2	[52]
2022	Interference	Tapered multi-core fiber	-	0.25–4.0	[53]
2022	FPI	Microsphere coupled off-core fiber	-	0.1–45.6	[54]
2023	FPI	TPP Technology	889 times that of the commercial sensor	0.052–0.384	[55]
2023	FPI	Hollow-core fiber	0.0068 nm/V	≤1.0	[56]
2023	FBG	Suspended polymer micro-rod	-	≤7.6	[57]
2024	FPI	Thin-walled microsphere	-	0.3–3.0	[58]
2024	FPI	Dual-resonant cavity	26.9 μPa/Hz^1/2^	0.1–2.25	[59]
2024	FPI	Hollow-core fiber	3.603 mV/kPa	≤5.4	[60]
2024	FPI	Two-photon polymerization 3D printing	23 Pa/Hz^1/2^	1.0–20.0	[61]
2025	FBG	Two-segment nonuniform fiber Bragg gratings	1.313 V/kPa	≤0.3	[62]
2025	FBG	PSFBG	-	≤0.513	[63]

**Table 4:** Center frequency and photoacoustic conversion efficiency of related research on photoacoustic materials in the past ten years.

Year	Materials	Center Frequency (MHz)	η (×10^−2^)	Reference
2015	CSNP	10	0.441	[69]
2018	CNTs-yarn	11.8	2.74	[70]
2020	CNT array	20.2	0.251	[71]
2021	MAPbI_3_	29.2	2.97	[72]
2023	MXene	8.06	1.25	[73]
2024	PDMS	~10	37.5 times than that of CNT	[74]
2025	MXene	17.4	1.21	[75]

**Table 5:** Microstructures and photoacoustic materials of FOPT in recent years.

Year	Microstructures	Photoacoustic Material	Reference
2009	Polishing	Graphite and epoxy	[76]
2020	Laser generated void	Gold and Cotronics 952 bonder	[77]
2023	TFBG	Multiwalled carbon nanotubes and PDMS	[78]
2024	LPFG	Graphite and epoxy	[79]
2024	Peanut-shaped	Graphite and epoxy	[28]
2025	Thermal cavitation effect	Cu(NO_3_)_2_ solution	[80]
2025	CPCF	Graphite and epoxy	[81]
2025	Peanut-shaped	Graphene and epoxy	[82]
2025	L-shaped offset splicing	MXene and PDMS	[83]

**Table 6:** The recent research on distributed FOPT arraying methods.

Year	Method	Multiplexing Number	Reference
2009	Side wall leakage	5	[76]
2013	Grating structures	3	[90]
2017	Tapering or collapsing	4	[89]
2017	Core-offset	5	[85]
2018	Side wall leakage	4	[84]
2019	Splicing of different fiber	5	[86]
2024	Grating structures	3	[79]
2024	Splicing of different fiber	3	[87]
2024	Splicing of different fiber	4	[88]
2025	Tapering or collapsing	3	[81]
2025	Core-offset	6	[82]
2025	Core-offset	4	[83]
2025	Multi-channel	4	[91]

**Table 7:** The recent research achievements in DPF-NDT imaging algorithms.

Year	Method	Features	Reference
2019	TFM	Matching pursuit with time-reversal focusing	[92]
2021	AI-Based	Based on genetic algorithm	[93]
2022	RAPID	Combined correlation-based damage indices with time-delay difference correction probability distribution functions	[94]
2022	TFM	Waveform correlation coefficients	[95]
2022	TFM	TR-MUSIC and PC-MUSIC	[96]
2023	TFM	Baseline-free and compressed sensing	[97]
2024	TFM	Baseline-free and path amplitude-matched	[98]
2024	AI-Based	Unsupervised algorithm using automatic gating and autoencoders	[99]
2025	RAPID	Using hybrid Copula functions	[100]
2025	RAPID	NERAPID	[101]
2025	AI-Based	support vector machine	[102]

**Table 8:** The applications of DFOPTA in recent years.

Year	Application	Reference
2018	Steel rebar corrosion	[84]
2023	Aluminum defect	[28]
2025	Aluminum and CFRP defect	[81]
2025	Aluminum defect	[82]
2025	Aluminum defect	[83]
2025	CFRP defect	[91]

## Data Availability

The original contributions presented in this study are included in the article. Further inquiries can be directed to the corresponding author.

## Abbreviations

AI	artificial intelligence
ARROW	anti-resonant reflecting optical waveguide
CFRP	carbon fiber reinforced polymers
CPCF	collapsed photonic crystal fiber
CSNP	candle soot nanoparticle
DFOPTA	distributed fiber-optic photoacoustic transducer array
DFP-NDT	distributed fiber-optic photoacoustic non-destructive testing
DFPS	distributed fiber-optic photoacoustic sensing
FBG	fiber Bragg grating
FOPT	fiber-optic photoacoustic transducer
FPI	Fabry–Pérot interferometer
FSPSFBG	femtosecond phase-shifted fiber Bragg grating
FWHM	full width at half maximum
FZP	Fresnel zone plate
HCF	hollow-core fiber
LPFG	long-period fiber grating
MEMS	microelectromechanical systems
MoS_2_	molybdenum disulfide
NDT	non-destructive testing
NEP	noise-equivalent pressure
NE-RAPID	nonlinear-exponentially weighted RAPID
PAM	micron-resolution photoacoustic microscopy
PC-MUSIC	phase-coherent imaging with multiple signal classification
PDMS	polydimethylsiloxane
PE	polyethylene
PZT	piezoelectric transducer
RAPID	reconstruction algorithm for probabilistic inspection of damage
SMF	single-mode fiber
SMS	single-mode-multimode-single-mode
SMTC	single-mode-multimode-thin-cladding
SNR	signal-to-noise ratio
STA/LTA	short-term average/long-term average
TFBG	tilted fiber Bragg grating
TFCF	taper diameter on a four-core fiber
TFM	total focusing method
TNFBG	two-segment non-uniform fiber Bragg grating
TPP	two-photon polymerization
TR-MUSIC	time-reversal imaging with multiple signal classification
UT	ultrasonic testing
WDM	wavelength-division multiplexed
